# Hypoxia protects H9c2 cells against Ferroptosis through SENP1-mediated protein DeSUMOylation

**DOI:** 10.7150/ijms.50804

**Published:** 2021-02-04

**Authors:** Yu-Ting Bai, Feng-Jun Xiao, Hua Wang, Ri-Li Ge, Li-Sheng Wang

**Affiliations:** 1 Qinghai Provincial People's Hospital, Xining, 810001, PR China.; 2 Beijing Institute of Radiation Medicine, Beijing, 100850, PR China.; 3 Research Center for High Altitude Medicine, Qinghai University, Xining, 810001, PR China.; 4 Department of Molecular Diagnosis and Regenerative Medicine, Medical Research Center, the Affiliate Hospital of Qingdao University, Qingdao 266000, PR. China.

**Keywords:** Ferroptosis, Hypoxia, SENP1

## Abstract

Hypoxia affects proliferation, differentiation, as well as death of cardiomyocyte, and plays an important role in the development of myocardial ischemia. However, the detailed mechanisms through which hypoxia regulates cardiomyocyte ferroptosis have not been explored. In this study, we revealed that hypoxia suppresses the proliferation, migration, and erastin-induced ferroptosis of H9c2 cells. First, we confirmed the upregulation of SENP1 in H9c2 cells cultured under hypoxic conditions. Through adenovirus-mediated SENP1 gene transfection, we demonstrated that SENP1 overexpression could enhance H9c2 cell proliferation and migration while also protecting H9c2 cells from erastin-induced ferroptosis. Furthermore, through immunoprecipitation and western blotting, we confirmed that SENP1 mediated deSUMOylation of HIF-1α and ACSL4 in H9c2 cells. In conclusion, this study describes the underlying mechanism through which hypoxia upregulates SENP1 expression, in turn protecting against ferroptosis via the regulation of HIF-1α and ACSL4 deSUMOylation. Our findings provide a theoretical foundation for the development of novel therapeutics for ischemic heart diseases.

## Introduction

Cardiomyocyte death, which causes progressive cell loss and subsequent heart failure, is the most important pathological characteristic of heart diseases 1. Several types of cell death, such as apoptosis, necrosis, autophagic cell death, and ferroptosis, have been reported to be involved in myocardial ischemia 2, 3. Hypoxia is implicated in various pathologies of the heart. However, the detailed mechanisms through which hypoxia regulates cardiomyocyte death have not been extensively explored.

Ferroptosis is a newly described form of regulated cell death, which was defined by Dixon in 2012 4. It is iron-dependent and distinct from apoptosis, necroptosis, and other types of cell death 5. Ferroptosis is implicated in various conditions, such as ischemia, tumors, and tissue injuries 6-8. It has been reported that ferroptosis occurs in ischemic cardiomyocytes and may cause cell loss, and subsequent heart failure 6, 9. Targeting ferroptosis conveys protection against cardiomyopathy in doxorubicin- and ischemia/reperfusion (I/R)-induced murine models 10. However, the exact effect of hypoxia on cardiomyocyte ferroptosis in the heart remains unclear.

Several regulatory molecules, including glutathione peroxidase 4 (GPX4) 11, acyl-CoA synthetase long-chain family member 4 (ACSL4) 12, p53 13, and phosphatidylethanolamine binding protein 1 (PEBP1) 14, have been reported to be involved in the execution of ferroptosis. ACSL4 is an isozyme that belongs to the long-chain fatty-acid-coenzyme A ligase family. ACSL4 regulates ferroptosis sensitivity by modulating cellular lipid composition 12. Given the important roles of ACSL4 in ferroptosis, its regulatory role in cardiomyocyte ferroptosis requires further research in order to improve our understanding of hypoxia and heart diseases.

SUMOylation is a reversible post-translational modification characterized by the binding of small ubiquitin-like modifiers (SUMOs) to target proteins in eukaryotic cells 15. SUMOylation regulates the activities of multiple signaling proteins and enzymes with critical roles in a variety of cellular processes, such as DNA replication and repair, genome integrity, nuclear transport, signal transduction, and cell proliferation 16. Sentrin-specific protease 1 (SENP1) is a human protease that deconjugates SUMO molecules from SUMOylated proteins and regulates SUMO-related pathways 17. Aberrant SENP1 expression and activation have been reported to be associated with various malignancies, including prostate cancer, lung cancer, multiple myeloma, and colon cancer 18-21.

Accumulating evidence has revealed that SENP1 functions as a hypoxia- responsive gene. SENP1 promotes hypoxia-induced cancer stemness by inducing hypoxia-inducible factor 1 alpha (HIF-1α) deSUMOylation and a SENP1/HIF-1α positive feedback loop 22. The SENP1/HIF-1α feedback modulates hypoxia-induced proliferation, invasion, and epithelial-mesenchymal transition (EMT) of tumor cells 23. However, regulatory roles and mechanisms of SENP1 in the hypoxic response and ferroptosis have not yet been explored. In this study, we aimed to clarify the role of SENP1-mediated protein deSUMOylation in H9c2 cell ferroptosis in order to determine whether it is a potential therapeutic target for ischemic heart diseases.

## Results

### Hypoxia suppresses the proliferation and migration of H9c2 cells

**Figure 1:**
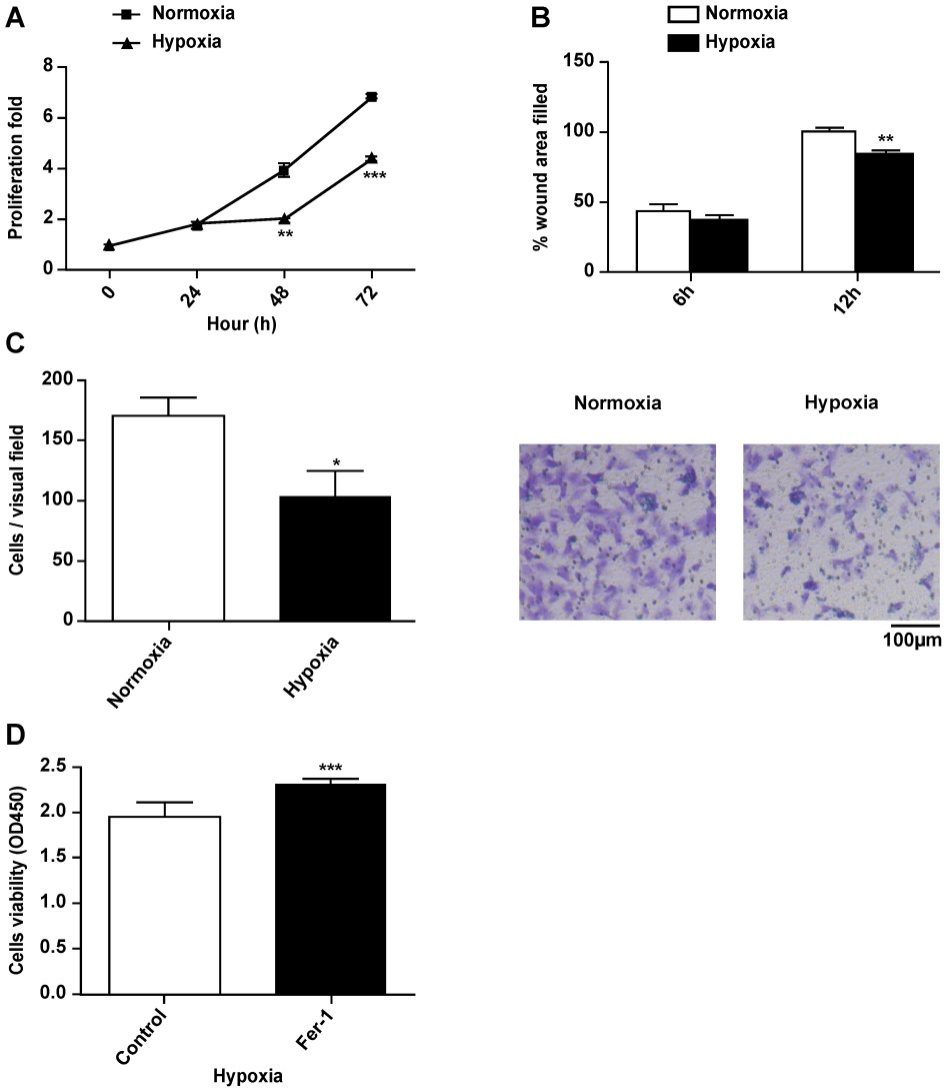
** Hypoxia suppresses proliferation and migration of H9c2 cells. (A)** H9c2 cells were incubated in a hypoxic incubator under 1% O_2_, 5% CO_2_, and 94% N_2_. Cell proliferation was determined via the CCK-8 assay for 72h and shown as the fold increase compared to 0 h. **(B)** Confluent H9c2 cells were scratched to generate wounds. The percentages of wound area filled were measured and analyzed. **(C)** The migratory ability was analyzed using a Transwell assay. Representative images and the statistical results are shown, magnification: 20×. **(D)** The cells were cultured under hypoxic condition in the absence or presence of Fer-1 (1μM). Cell viability was determined via the CCK-8 assay. All experiments were repeated at least three times, and data are presented as the mean ± SD. *p < 0.05, **p < 0.01, ***p < 0.001 vs. the control group.

To explore the effects of hypoxia on the characteristics of H9c2 cells, we cultured H9c2 cells in a hypoxic incubator containing 1% O_2_, 5% CO_2_ and 94% N_2_. The proliferation and migration of H9c2 cells under hypoxic and normoxic conditions were measured. Compared to the normoxia group, hypoxia significantly decreased the proliferation of H9c2 cells after 24 h (Fig. 1A). Hypoxia decreased the migratory ability of H9c2 cells in both wound healing and Transwell assays (Fig. 1B, C). There were no differences between the effects of short-term hypoxia and normoxia on cell proliferation and migration. However, after long exposure times, hypoxia suppressed both cardiomyocyte proliferation and migration. Ferrostatin-1 treatment rescued the hypoxia-induced inhibition of H9c2 cell proliferation (Fig. 1D), suggesting the involvement of Fe metabolism and ferroptosis in hypoxia-induced growth inhibition.

### Hypoxia protects H9c2 cells from ferroptosis and inhibits erastin-induced ROS generation

**Figure 2:**
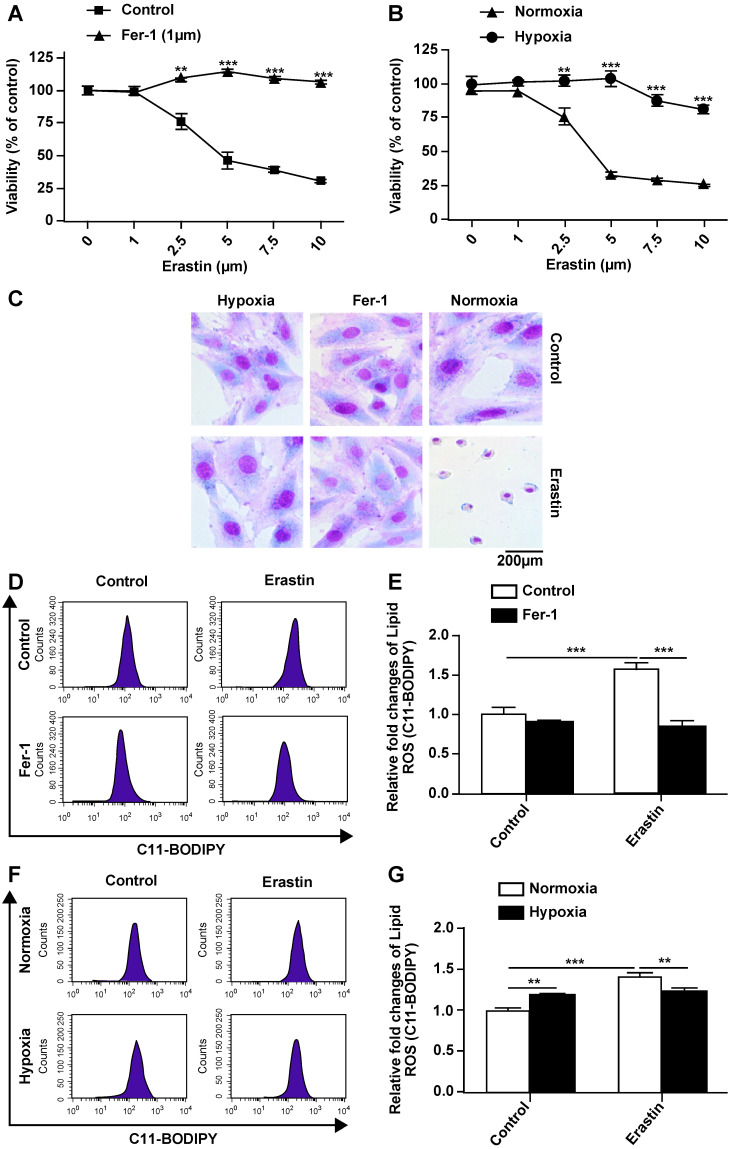
** Hypoxia inhibits ferroptosis of H9c2 cells. (A)** Effect of Fer-1 (1 μM) on the lethality of erastin in H9c2 cells after treatment for 24 h. **(B)** Effect of hypoxia on the lethality of erastin in H9c2 cells after treatment for 24 h. **(C)** Morphological changes of H9c2 cells after treatment with erastin (2.5 μM) under hypoxic or normoxic conditions, magnification: 40×. **(D, E)** Effect of Fer-1 (1 μM) on erastin (5 μM)-induced ROS production in H9c2 cells. ROS production was measured by flow cytometry using C11-BODIPY581/591. Representative images and the statistical results are shown. **(F, G)** Effect of hypoxia on erastin (5 μM)-induced ROS production in H9c2 cells. Representative images and the statistical results are shown. All experiments were repeated at least three times, and data are presented as the mean ± SD. **p < 0.01, ***p < 0.001 vs. the control group at the same time point.

Erastin is an experimentally verified agent for the induction of ferroptosis 4. We previously confirmed that erastin is a typical inducer of ferroptosis in H9c2 cells. In the present study, erastin-induced ferroptosis of H9c2 cells under hypoxic conditions was assessed. In particular, we tested whether hypoxia can change the sensitivity of H9c2 cells to erastin-induced ferroptosis. Hypoxia suppressed erastin-induced H9c2 cell death (Fig. 2B). Further, erastin-induced cell death was reversed by ferrostatin-1 in a dose-dependent manner (Fig. 2A). Giemsa staining of erastin-treated cells revealed typical ferroptotic morphological characteristics, such as cell volume shrinkage (Fig. 2C). Ferrostatin-1 and hypoxia both protected the cells from ferroptosis. Thus, we assessed ROS accumulation following erastin treatment under normoxia and hypoxia. Lipid ROS levels were increased after the erastin treatment, and ROS accumulation was suppressed by co-treatment with ferrostatin-1 in H9c2 cells under normoxia (Fig. 2D, E). Hypoxia protected H9c2 cells against erastin-induced lipid ROS accumulation (Fig. 2F, G). Cumulatively, these results suggested that hypoxia protects H9c2 cells from erastin-induced ferroptosis by preventing ROS accumulation.

### Hypoxia upregulates SENP1 and ferroptosis-related gene expression in H9c2 cells

**Figure 3:**
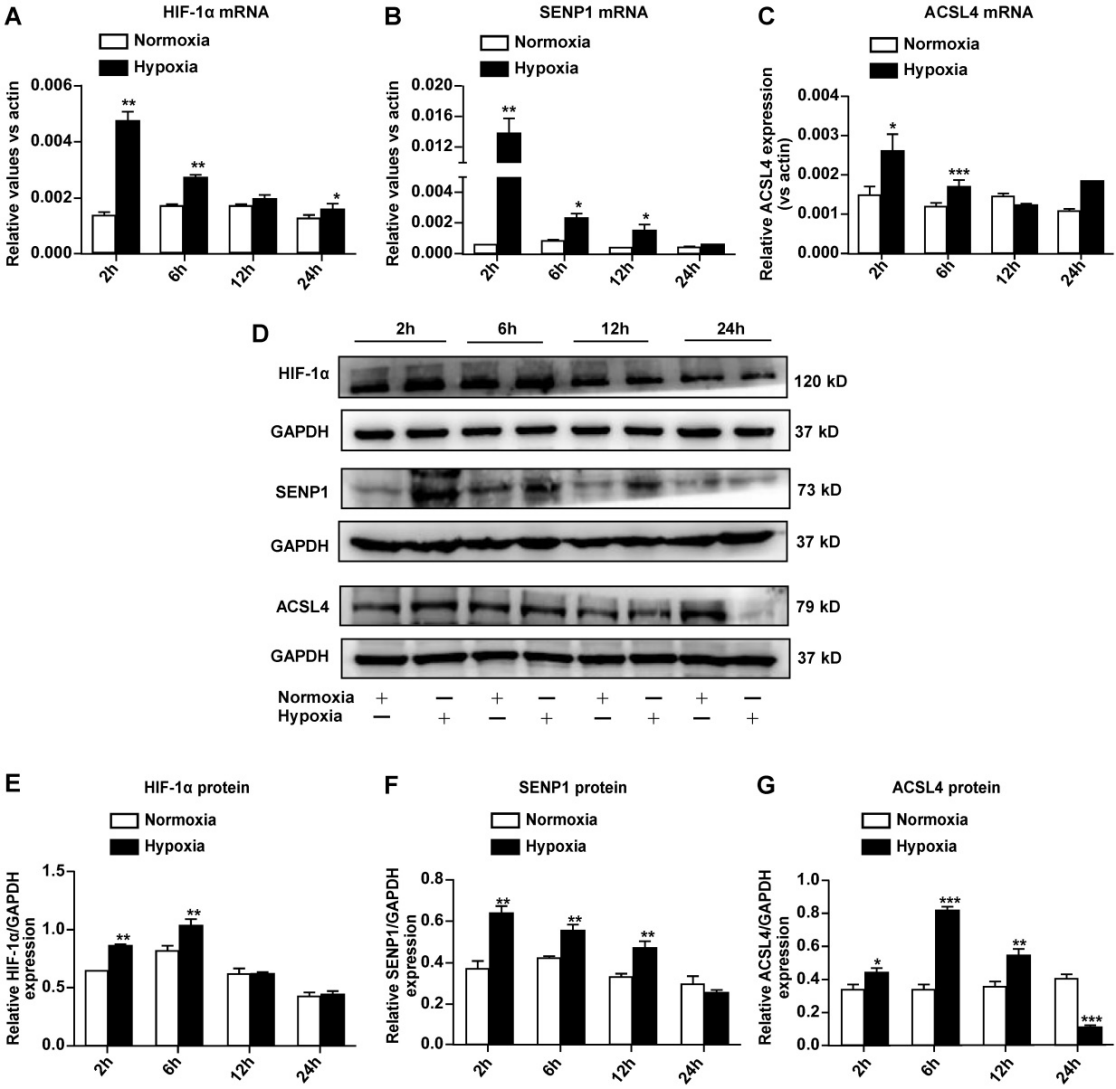
** Hypoxia regulates SENP1 and ferroptosis-related gene expression in H9c2 cells. (A-C)** Total RNA was isolated and the mRNA expression of HIF-1α (A), SENP1 (B), and ACSL4 (C) was analyzed by qRT-PCR after culturing under hypoxic conditions for 2 h, 6 h, 12 h, and 24 h. **(D)** Western blot analysis was used to measure the protein levels of HIF-1α, SENP1, and ACSL4 under hypoxic conditions for 2 h, 6 h, 12 h, 24 h. **(E-G)** Western blot densitometry analysis of HIF-1α (E), SENP1 (F), and ACSL4 (G). All experiments were repeated at least three times, and data are presented as the mean ± SD. *p < 0.05, **p < 0.01, ***p < 0.001 vs. the control group.

To further elucidate the mechanisms through which hypoxia suppresses ferroptosis in H9c2 cells, we examined the expression of hypoxia-responsive genes such as HIF-1α and SENP1, as well as ferroptosis-related ACSL4. Quantitative RT-PCR revealed that the mRNA expression of HIF-1α (Fig. 3A), SENP1 (Fig. 3B), and ACSL4 (Fig. 3C) increased after hypoxia exposure. Western blot analysis indicated that HIF-1α (Fig. 3D, E), SENP1 (Fig. 3D, F), and ACSL4 (Fig. 3D, G) protein levels also increased under hypoxia. Upregulation of these genes occurred during the early stage of hypoxia exposure, and expression returned to basal levels within 24 h. ACSL4 was an exception, as its expression decreased after hypoxia exposure for 24 h. Based on the above-described results, SENP1 and ACSL4 were confirmed as hypoxia-responsive genes.

### SENP1 overexpression promotes proliferation and migration of H9c2 cells

**Figure 4:**
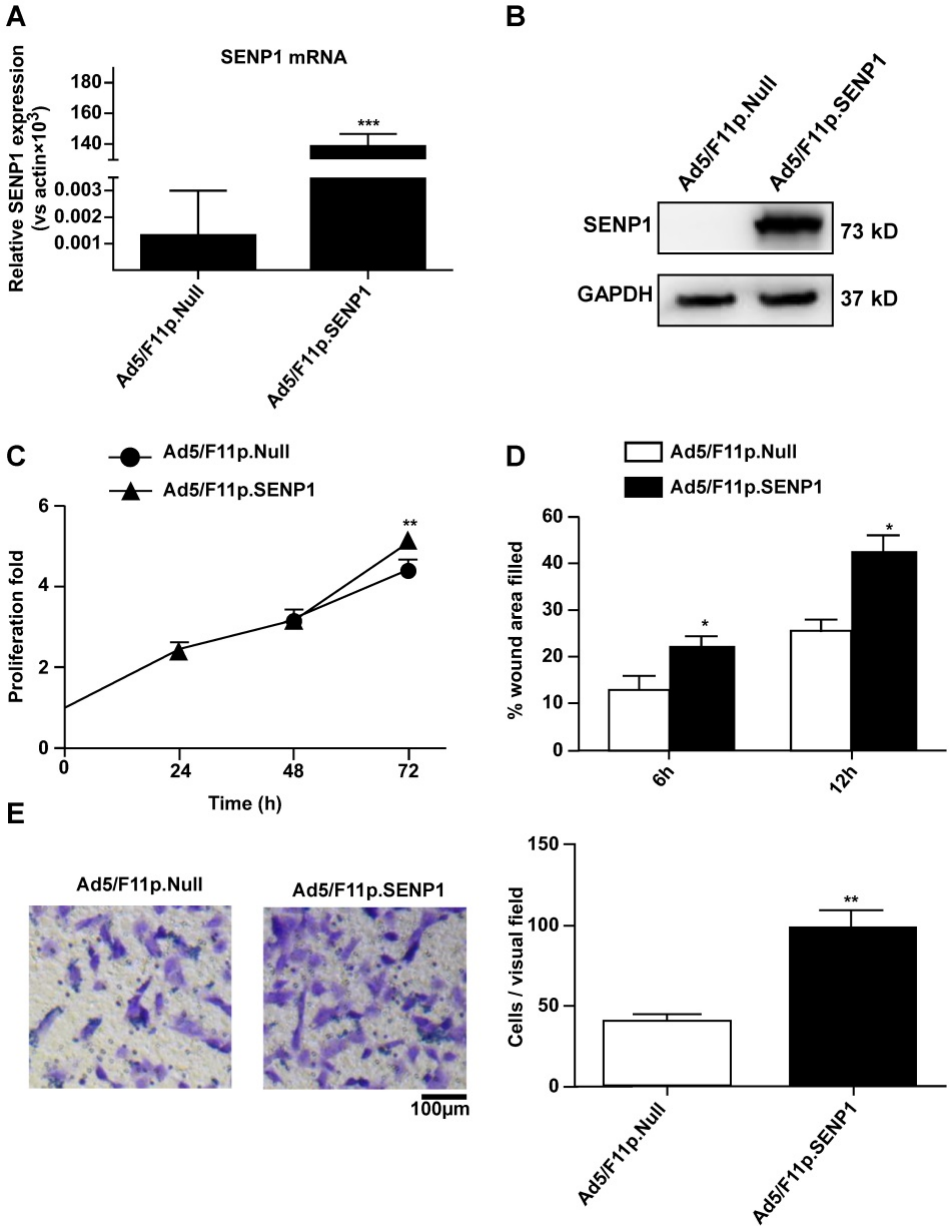
** SENP1 overexpression promotes proliferation and migration of H9C2 cells. (A)** Adenovirus-mediated overexpression of SENP1 in H9c2 cells. H9c2 cells were infected with 20MOI of Ad5/F11p.SENP1 or Ad5/F11p.Null. After 24 h of infection, the total RNA was isolated, and the mRNA expression of SENP1 was determined via qRT-PCR. **(B)** After 48 h of infection, the SENP1 protein expression was detected via western blot analysis. **(C)** The H9c2 cells were cultured for 72 h. Cell proliferation was determined via the CCK-8 assay and is presented as the fold increase compared to 0 h. **(D)** Confluent H9c2 cells were scratched to generate wounds, at 24 h after infection with adenovirus. The percentages of wound area filled were determined and analyzed. **(E)** Migratory ability was analyzed via the Transwell assay at 24 h after transfection with Ad5/F11p.SENP1. Representative images and the statistical results are shown, magnification: 20×. All experiments were repeated at least three times, and data are presented as the mean ± SD. *p < 0.05, **p < 0.01, ***p < 0.001 vs. the Ad5/F11p.Null group at the same time point.

To explore the role of SENP1 in regulating H9c2 cell characteristics, we constructed an adenoviral vector (Ad5/F11p.SENP1) in order to overexpress the SENP1 gene. Transduction of H9c2 cells with Ad5/F11p.SENP1 upregulated SENP1 expression at both the mRNA (Fig. 4A) and protein (Fig. 4B) levels. SENP1 overexpression significantly promoted cell proliferation (Fig. 4C). We also assessed the migration of SENP1-overexpressing cells. SENP1 overexpression enhanced the migratory ability of H9c2 cells in both the wound healing and Transwell assays (Fig. 4D, E). These results indicated the role of SENP1 in the regulation of H9c2 cell proliferation and migration.

### SENP1 overexpression suppresses ferroptotic cell death of H9c2 cells

**Figure 5:**
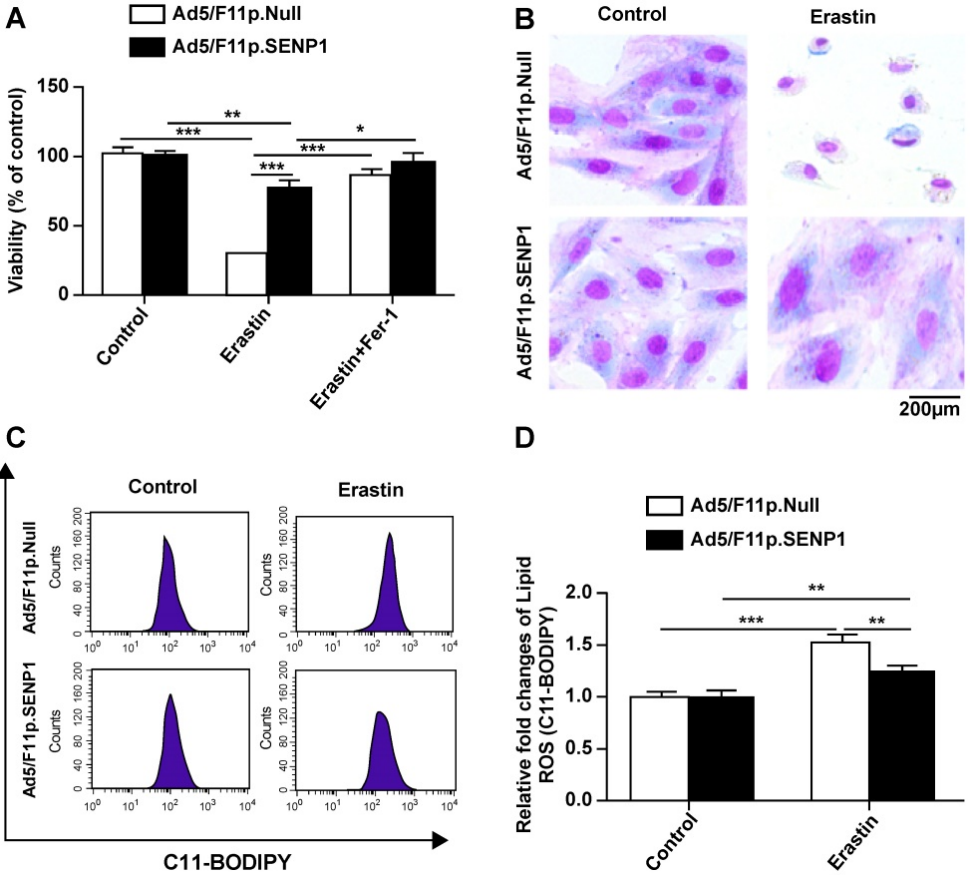
** SENP1 overexpression inhibits ferroptosis of H9c2 cells. (A)** Response of H9c2 cells transfected with Ad5/F11p.SENP1 or Ad5/F11p.Null to erastin (2.5 μM) ± Fer-1 (1 μM). **(B)** Morphological changes in H9c2 cells transfected with Ad5/F11p.SENP1 or Ad5/F11p.Null and then treated with erastin (5 μM), magnification: 40×. **(C, D)** Lipid ROS in erastin-treated H9c2 cells were measured using C11-BODIPY staining and flow cytometry. Representative images (C) and the statistical results (D) are shown. All experiments were repeated at least three times, and data are presented as the mean ± SD. *p < 0.05, **p < 0.01, ***p < 0.001 vs. the Ad5/F11p.Null group.

We then assessed the effects of SENP1 on ferroptotic cell death and observed that overexpression attenuated erastin-induced cell death (Fig. 5A). The morphology of SENP1-overexpressing cells also indicated less ferroptosis-associated changes compared to control cells (Fig. 5B). To assess the effects of SENP1 on ROS accumulation, we measured ROS production in H9c2 cells using C11-BODIPY581/591 staining. We found that SENP1 overexpression significantly inhibited ROS production in H9c2 cells treated with erastin (Fig. 5C, D). These findings suggested that SENP1 overexpression prevented ferroptosis, at least in part, by suppressing ROS production.

### SENP1 regulates HIF-1α deSUMOylation

**Figure 6:**
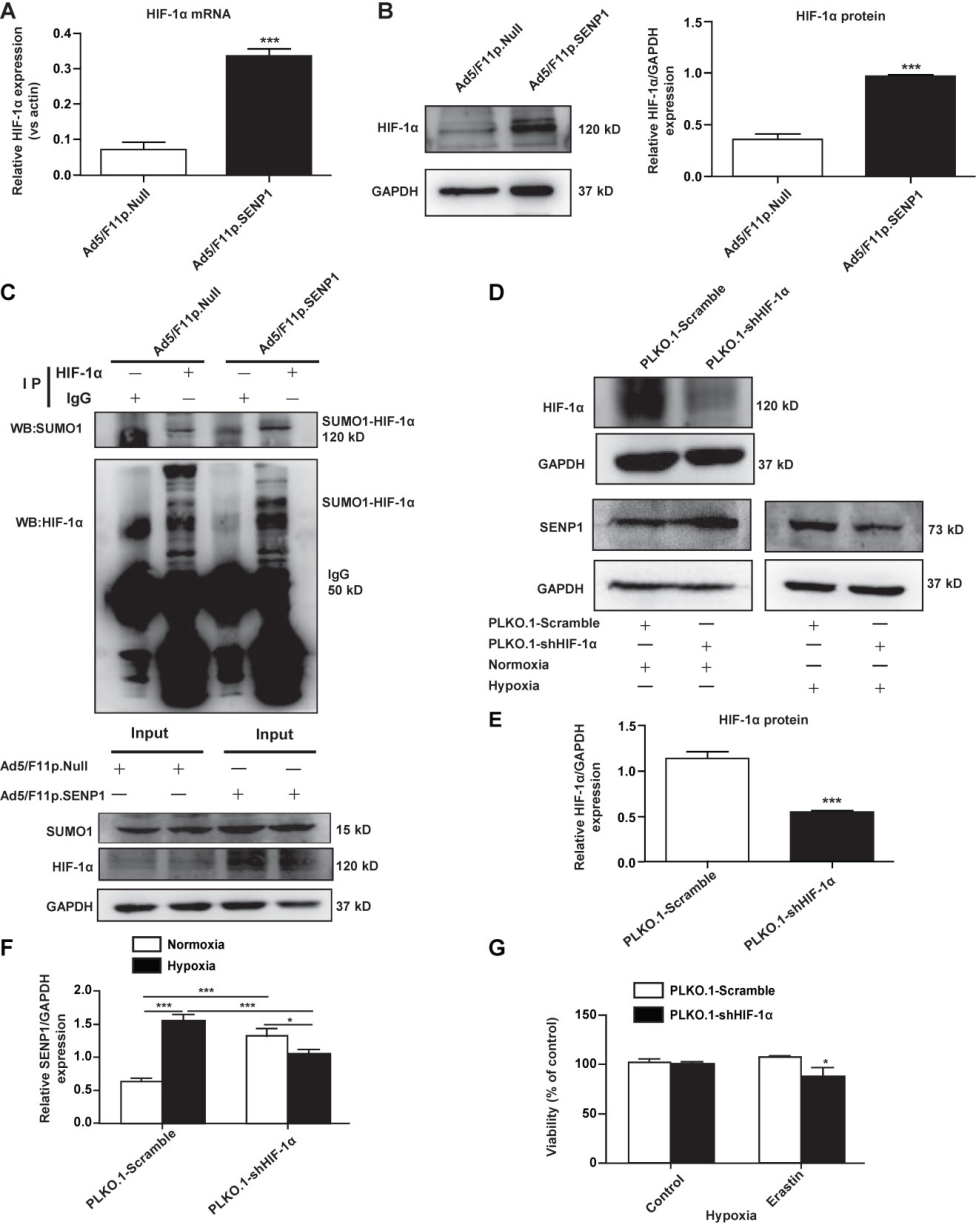
** SENP1 deSUMOylates HIF-1α and promotes its expression. (A, B)** H9c2 cells were infected with Ad5/F11p.SENP1 or Ad5/F11p.Null for 48 h, and mRNA and protein expression of HIF-1α was analyzed via qRT-PCR and western blot, respectively. **(C)** After transfection with Ad5/F11p.SENP1 or Ad5/F11p.Null, cells were collected for immunoprecipitation analysis. Representative images are shown. **(D, E)** H9c2 cells were transfected with PLKO.1-shHIF-1α or a control plasmid for 48 h. Protein expression of HIF-1α was analyzed via western blot (D). Densitometry analysis for HIF-1α (E). **(D, F)** After transfection with PLKO.1-shHIF-1α or a control plasmid, protein expression of SENP1 under normoxia or hypoxia was analyzed via western blot (D). Densitometry analysis of SENP1 (F). **(G)** Knockdown of HIF-1α inhibited the protective effects of hypoxia against erastin-induced cell death. All experiments were repeated at least three times, and data are presented as the mean ± SD. *p < 0.05, ***p < 0.001 vs. the control group.

To test whether SENP1 can deSUMOylate HIF-1α, we analyzed HIF-1α expression in H9c2 cells transfected with Ad5/F11p.SENP1 or Ad5/F11p.Null. We found that SENP1 overexpression upregulated HIF-1α at both the mRNA level (Fig. 6A) and protein levels (Fig. 6B). Through immunoprecipitation (IP), we confirmed that SENP1 directly deSUMOylated HIF-1α to alter its levels (Fig. 6C). In order to investigate whether the SENP1 expression was regulated by HIF-1α, we examined SENP1 protein levels after HIF-1α shRNA transduction. SENP1 expression was markedly increased in HIF-1α shRNA-transfected H9c2 cells under normoxia. These effects were abolished when HIF-1α was knocked down under hypoxia (Fig. 6D-F), indicating that HIF-1α silencing could upregulate SENP1 expression via a feedback mechanism. Furthermore, HIF-1α inhibition enhanced erastin-induced ferroptosis of H9c2 cells (Fig. 6G).

### SENP1 regulates ACSL4 during erastin-induced ferroptosis of H9c2 cells

**Figure 7:**
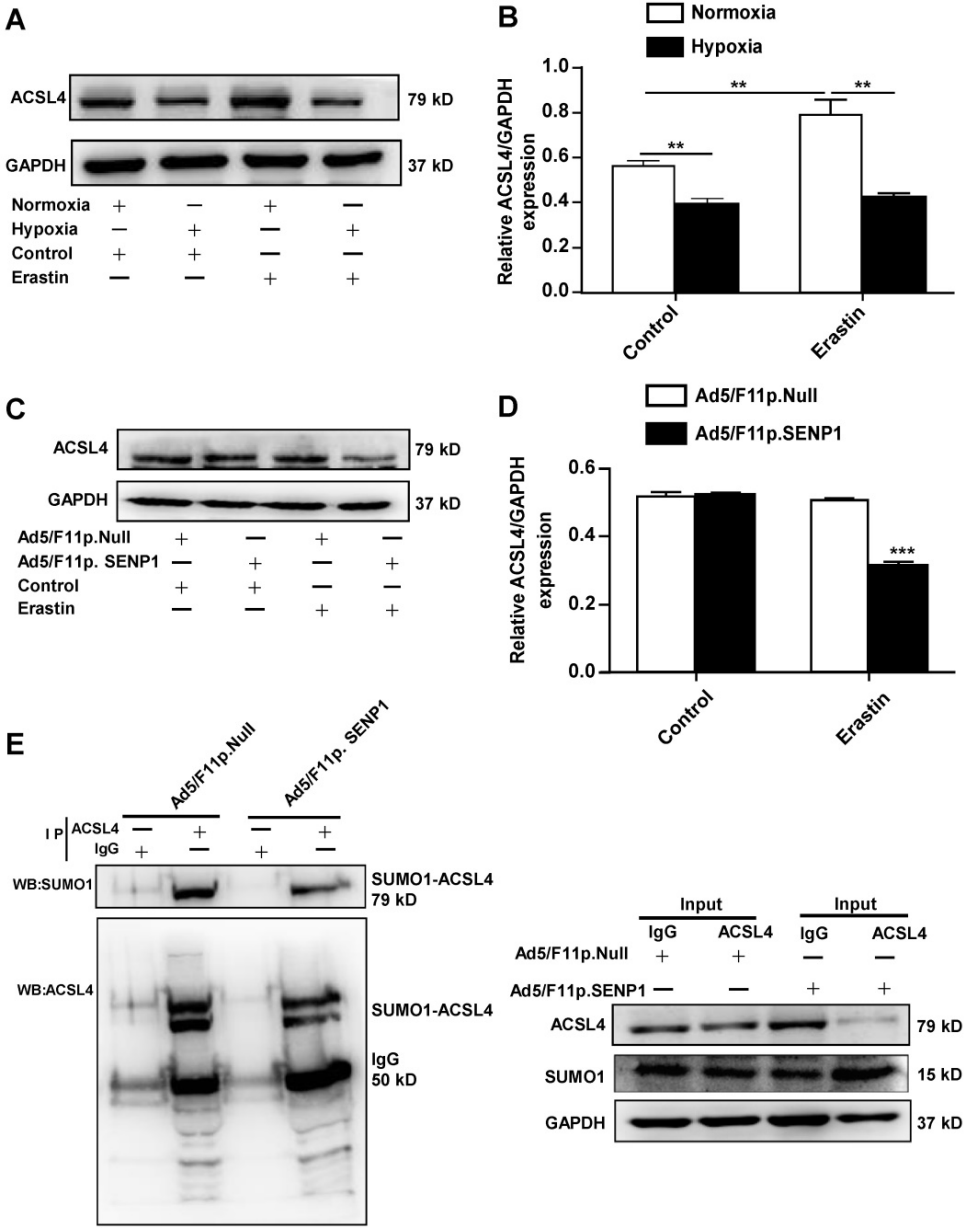
** SENP1 deSUMOylates ACSL4 and regulates its expression. (A, B)** Cells were treated with erastin (5 μM) and then incubated under hypoxia or normoxia. After 24 h, ACSL4 protein levels were detected via western blot (A). Densitometry analysis of ACSL4 (B). **(C, D)** H9c2 cells were infected with 20 MOI of Ad5/F11p.SENP1 or Ad5/F11p.Null. After 24 h, cells were treated with erastin (5 μM) and incubated under hypoxia for another 24 h. The protein levels of ACSL4 were detected via western blot (C). Densitometry analysis of ACSL4 (D). **(E)** After transfection with Ad5/F11p.SENP1 or Ad5/F11p.Null, cells were collected for immunoprecipitation analysis. Representative images are shown. All experiments were repeated at least three times, and data are presented as the mean ± SD. *p < 0.05, **p < 0.01, ***p < 0.001 vs. the control group.

As ACSL4 is considered a driver of ferroptosis, we examined ACSL4 levels in erastin-treated cells under hypoxic and normoxia. The expression of ACSL4 was markedly increased in erastin-treated cells, whereas hypoxia inhibited erastin-induced ACSL4 upregulation (Fig. 7A, B). Erastin did not induce ACSL4 upregulation in SENP1-overexpressing H9c2 cells under hypoxic conditions (Fig. 7C, D). The SUMOylation of ACSL4 was confirmed by immunoprecipitation and western blotting. Furthermore, SENP1 overexpression influenced the SUMOylation levels of HIF-1α and ACSL4 (Fig. 7E). These data demonstrated that SENP1-mediated ACSL4 regulation is involved in erastin-induced cardiomyocyte ferroptosis.

## Discussion

Heart failure (HF) is the end stage of cardiovascular disease and is characterized by the loss of cardiomyocytes due to cell death 24. Accumulating evidence indicates that cell death is an early and critical event in hypoxia-induced heart failure. Hypoxia affects cardiomyocyte proliferation, differentiation, and death, playing an important role in myocardial ischemia. In this study, the effect of hypoxia on H9c2 cell ferroptosis was investigated, and the roles of SENP1-mediated protein deSUMOylation under hypoxia were elucidated.

Hypoxia has been reported to both suppress and promote cardiomyocyte cell death 25, 26. We found that hypoxia suppressed proliferation and migration, but protected H9c2 cells against ferroptosis. Interestingly, hypoxia-induced growth inhibition could be reversed by ferrostatin-1, a lipophilic antioxidant that effectively blocks ferroptosis 27. To determine the effect of hypoxia on cardiomyocyte ferroptosis, we assessed the viability of H9c2 cells treated with erastin under hypoxic and normoxic conditions. To the best of our knowledge, the present study is the first to reveal that hypoxia could protect the H9c2 cells against erastin-induced ferroptosis. We also detected the expression of hypoxia- and ferroptosis-related molecules. Hypoxia increased HIF-1α and SENP1 expression in cardiomyocytes at the early stage of exposure. Thus, it was suggested that SENP1 might be involved in the hypoxia-induced survival of H9c2 cells.

Ferroptosis is an iron-dependent form of cell death, characterized by cell volume shrinkage and mitochondrial membrane thickening, and mediated by iron-dependent lipid peroxide accumulation. Ferroptosis has been reported in I/R injury and a number of other diseases 28. Several novel strategies and therapeutic agents have been shown to significantly improve heart function through the mitigation of cardiomyocyte ferroptosis 29. SENP1 plays important roles in the regulation of multiple cellular signaling pathways. It protects against myocardial I/R injury via the HIF-1α-dependent pathway 30. Hypoxia has been revealed as a critical inducer of SENP1 expression 31. However, the physiological role of SENP1 in hypoxia-induced H9c2 cell death has remained elusive. Previously, we confirmed that erastin-induced growth inhibition is an ideal model for cardiomyocyte ferroptosis 32. In the current work, our* in vitro* experiments confirmed that hypoxia induced SENP1 upregulation in H9c2 cells. By using adenovirus-mediated SENP1 gene overexpression, we demonstrated that SENP1 protected the H9c2 cells against erastin-induced ferroptosis. Ferroptosis is mediated by the redox-active metal Fe and ROS generation. Thus, the inhibitory effect of SENP1 overexpression on ferroptosis was further confirmed by the blockage of erastin-induced ROS generation in H9c2 cells. Furthermore, the roles of SENP1 in H9c2 cell proliferation and migration were elucidated by CCK8 and Transwell assays. We demonstrated that SENP1 overexpression significantly increased the proliferation and migration of H9c2 cells. This is consistent with previous studies reporting that SENP1 regulates the proliferation and migration of other cell types 33, 34.

SUMOylation may affect H9c2 cell ferroptosis by modulating ferroptosis regulators, such as HIF-1α, ACSL4, and GPX4. Among these, ACSL4 acts as an essential driver of ferroptosis and is thus used as a ferroptosis biomarker. ACSL4 expression is remarkably downregulated in ferroptosis-resistant cells 35. Multiple signals, such as the α6β4-mediated activation of Src and STAT3, suppress the expression of ACSL4 36. In addition, pharmacological ACSL4 inhibition prevents ferroptosis 12. We found that hypoxia induced SENP1 expression, and SENP1 overexpression mediated deSUMOylation of HIF-1α and ACSL4. Post-translational modification of proteins with SUMO moieties is a tightly regulated and highly dynamic process. Thus, the regulation of ACSL4 through SUMOylation may play an important role in hypoxia-regulated signaling.

HIF-1α SUMOylation affects the stability and transcriptional activity of HIF-1α in several types of cells. It is known that SENP1 deconjugates SUMO from HIF-1α to regulate its protein level and functional activity, potentially contributing to leukemogenesis and chemoresistance 37. The SENP1/HIF-1α positive feedback loop mediates hypoxia-induced stemness in cancer cells 22. In relation to this feedback loop, our data indicated that SENP1 overexpression upregulated HIF-1α at the mRNA level, and HIF-1α shRNA transduction caused SENP1 upregulation. However, knockdown of HIF-1α only partially suppressed SENP1 expression under hypoxic conditions. Based on these results, it can be inferred that SENP1 is able to regulate ferroptosis by reducing SUMOylation and enhancing the stability and transcriptional activity of HIF-1α under hypoxia. Previous studies have reported that SENP1 knockdown decreases HIF-1α expression in H9c2 cells by enhancing SUMOylation, thereby resulting in enhanced cellular necrosis and apoptosis 30. To our knowledge, the present study is the first to report that SENP1 was involved in ferroptosis via the regulation of HIF-1α deSUMOylation under hypoxia.

As a major activator of ferroptosis, ACSL4 is required ferroptosis induction. We showed that erastin-induced ACSL4 upregulation was abolished by hypoxia and SENP1 overexpression. Through immunoprecipitation and western blotting, we confirmed the direct interaction between ACSL4 and SUMO1 molecules in H9c2 cells. SENP1 is likely to deconjugate SUMO1 from ACSL4, leading to a decrease in the ACSL4 protein levels in H9c2 cells under hypoxic conditions. However, erastin did not induce a significant increase in ACSL4 expression in SENP1-overexpressing H9c2 cells. ACSL4 downregulation induced by SENP1-mediated deSUMOylation might contribute to the protective effects of hypoxia against cell ferroptosis.

In conclusion, this study elucidated the mechanism through which hypoxia upregulates SENP1 expression, in turn protecting against H9c2 cells ferroptosis by regulating HIF-1α and ACSL4 levels. The findings of this study provide a theoretical basis for the future development of novel therapeutics for ischemic heart disease.

## Materials and Methods

### Cell culture

Rat embryonic cardiomyoblasts (H9c2 cell line) were obtained from the Institute of Basic Medical Sciences, Chinese Academy of Medical Sciences (Beijing, China) and cultured in Dulbecco's Modified Eagle's Medium (Gibco, Carlsbad, CA, USA) containing 10% FBS, 100 U/mL penicillin, and 100μg/mL streptomycin (Hyclone, Logan, UT, USA) and cultured at 37 °C under 5% CO_2_. To stimulate hypoxia, cells were cultured in a hypoxic incubator containing 1% O_2_, 5% CO_2_, and 94% N_2_.

### RNA extraction and quantitative RT-PCR

Total RNA was extracted from cells using the TRIzol reagent (Invitrogen Life Technologies, Carlsbad, CA, USA) and cDNA was synthesized using the RevertAidTM First Strand cDNA Synthesis Kit (Thermo Scientific, Wilmington, DE) according to the manufacturer's instructions. qPCR was performed to amplify the target cDNA by using the SYBR® Premix Ex Taq kit (TaKaRa, Dalian, China). β-actin was used an internal control to normalize target mRNA levels. The PCR primers were designed and synthesized by Tsingke (Beijing, China). Primer sequences were as follows: SENP1-forward: 5'-AGAGTCCAGTTCACAGTTCCAT-3'; SENP1-reverse: 5'-CACGGCAGCAGGTAGGATAA-3'; β-actin-forward: 5'-GGAGATTACTGCCCTGGCTCCTA-3'; β-actin-reverse: 5'-GACTCATCGTACTCCTGCTTGC TG-3'; HIF-1α-forward: 5'-CCGAGTGTGAGCACAGTTACA-3'; HIF-1α-reverse: 5'-TTCATCAGTGGTGGCAGTTG-3'; ACSL4-forward: 5'-TGTATTTGAAGGGAGGCTGA AT-3'; ACSL4-reverse: 5'-CTCAGCCTTTGACTAGATCACA-3'.

### Vectors

Chemically fiber-modified and replication-deficient adenovirus expressing SENP1 (Ad5/F11p.SENP1, constructed in our laboratory) and a control vector (Ad5/F11p.Null, constructed in our laboratory) were constructed as previously described 34. The recombinant plasmid PLKO.1-shHIF-1α (GTTACGTTCCTTCG ATCAG) and a control plasmid were kindly provided by Dr. Cui CP at the Beijing Institute of Radiation Medicine 22. For shRNA transfection, 10 μL of shRNA and 10 μL of Lipofectamine 3000 (Invitrogen, San Diego, CA, USA) were used, according to the manufacturer's instructions.

### Western blotting, wound healing assays, and measurement of ROS production

Western blotting, wound healing assays, and measurement of ROS production were performed as previously described 31.

### Cell viability assays

Cell viability was assessed using the CCK-8 (Dojindo Molecular Technologies, Kumamoto, Japan) assay. Cells were cultured in 96-well plates at 5000 cells/well and cultured at 37 °C. After treatment, 10 µL CCK8 solution was added into each well at 0 h, 24 h, 48 h, and 72 h. The mixture was then incubated for 2 h at 37 °C under 5% CO_2_. The absorbance at a wavelength of 450 nm was measured using a microplate reader.

### Cell migration

Cell migration was assessed using Transwell chambers. After treatment, 2×10^4^ cells were suspended in 200 μL of serum-free medium in the top chamber, and 600 μL of complete medium was added to the lower chamber. After culturing for 10 h, the membranes were stained with crystal violet.

### Immunoprecipitation and immunoblotting

Cells were extracted with ice-cold lysis buffer and PMSF. Anti-ACSL4 antibodies or anti-HIF-1α antibodies (Abcam, Cambridge, Britain) were added to 500 µL protein extracts and incubated overnight at 4 °C. Immune complexes were obtained by the addition of agarose beads, washed with wash buffer, and bound proteins were then eluted by boiling in 40 µL SDS reducing sample buffer for 5 min according to the manufacturer's protocol (Absin, Shanghai, China). Immunoprecipitated complexes of either SUMO-1, HIF-1α or ACSL4 were loaded on the same gels, which were run at the same time within the same electrophoresis unit and were then examined by immunoblotting as described above.

### Statistical analysis

The software package IBM SPSS Statistics (SPSS Inc, USA) was used for statistical analysis. Data are presented as the mean ± SD of at least three experiments. Differences between groups were evaluated using ANOVA or Student's *t*-tests. Statistical significance was set at *P* < 0.05.

## Abbreviations

I/R: ischemia/reperfusion
GPX4: glutathione peroxidase 4
ACSL4: acyl-CoA synthetase long-chain family member 4
PEBP1: phosphatidylethanolamine binding protein1
SUMOs: small ubiquitin-like modifiers
SENP1: sentrin-specific protease 1
HIF-1α: hypoxia-inducible factor 1 alpha
EMT: epithelial-mesenchymal transition
IP: immunoprecipitation
HF: heart failure
